# Social bonding through shared experiences: the role of emotional intensity

**DOI:** 10.1098/rsos.240048

**Published:** 2024-10-30

**Authors:** Victor Chung, Rocco Mennella, Elisabeth Pacherie, Julie Grezes

**Affiliations:** ^1^ Département d’Études Cognitives, Institut Jean-Nicod, École Normale Supérieure, Université PSL, EHESS, CNRS, Paris, France; ^2^ Département d’Études Cognitives, Laboratoire de Neurosciences Cognitives et Computationnelles (Inserm U960), École Normale Supérieure, Université PSL, Paris, France; ^3^ Laboratoire des Interactions Cognition, Action, Émotion (LICAÉ), Université Paris Nanterre, Nanterre, France

**Keywords:** collective emotion, shared experience, physiological synchrony, collective effervescence, social bonding, joint attention

## Abstract

Sharing emotions with other individuals is a widespread phenomenon. Previous research proposed that experiencing intense and similar emotions with other individuals reinforces social bonds. However, several aspects of this phenomenon remain unclear, notably whether social bonding requires the convergence and synchronization of emotions in the group, and whether these effects generalize across positively valenced and negatively valenced emotional contexts. To address these questions, we measured subjective emotional experiences, physiological activity (cardiac, respiratory, electrodermal) and social attitudes in dyads of unacquainted individuals who watched videos in the presence of each other. We manipulated the emotional content of the videos and the type of shared attention between participants, to test for the contribution of interpersonal influence. The results revealed that intense emotions indexed by physiological arousal were associated with the emergence of reciprocal prosocial attitudes within dyads, and that this effect depended on joint attention. We did not observe the convergence and synchronization of emotions within dyads, which suggests that experiencing similar emotions was not necessary for social bonding. We discuss implications of this study for research on collective effervescence and the social consequences of shared experiences.

## 1. Introduction

Over a century ago, the sociologist Émile Durkheim [1] observed that intense emotional events reinforce social bonds between group members. He hypothesized that social rituals elicit collective effervescence, which is a ‘shared emotional state of high intensity’ [2, p. 16] that emerges from the synchronization of attention and behaviour in the group. Since then, researchers have applied the concept of collective effervescence to social protests (e.g. [3]), sporting events (e.g. [4]), live artistic performances (e.g. [5]) and everyday social interactions [6] such as watching movies together [7]. A meta-analysis of studies on collective effervescence supported Durkheim’s hypothesis [8]: participants in social gatherings who experienced higher emotional arousal reported feeling more connected to others and were more committed to their group. Therefore, research on collective effervescence provided evidence that emotion contributes to social bonding. However, several issues remain to be investigated. On the one hand, it is well known that emotion is a multifaceted phenomenon, comprising responses at several levels, including physiological responses [9]. Yet, in previous studies, emotion has mainly been measured by means of subjective reports (but see [5,10]). On the other hand, different lines of research have complemented the investigation of Durkheim’s hypothesis, suggesting that social bonding not only requires emotions to be intense but also shared with other group members, as reviewed below.

Shared emotion can be defined as an interpersonal pattern of emotions that emerges at group level [11–13]. Previous research reviewed several cognitive mechanisms that are responsible for patterns of emotional convergence (similarity in form and content) and synchronization (similarity in time course) [14–17]. Patterns of emotional similarity are interpersonal insofar as they emerge when individuals reciprocally influence each other during social interactions (i.e. interpersonal alignment [18,19]). Accordingly, shared emotion not only requires that individuals simultaneously experience the same event, but also that they experience the event together. This is closely related to the concept of ‘shared attention’: a minimal form of shared attention implies that individuals simultaneously allocate attention to the same point in space, whereas a higher form additionally requires that individuals be mutually aware thereof and can attend to each other, which is often defined as joint attention [20,21]. Crucially, previous studies in human and non-human primates revealed that joint visual attention promoted affiliative attitudes and behaviours [22–24]. Hereafter, we review experimental evidence showing that the effect of joint attention on social bonding varied with the emotions of participants and the similarity of emotions between participants.

On the one hand, previous research showed that the effect of joint attention on affiliative attitudes varied with emotion [25,26]. For example, participants attending videos on the same screen reported greater prosocial attitudes than participants attending videos on individual screens, although this effect was only observed in response to the most negative and arousing of the videos [25]. In contrast, another study found that participants felt more connected to each other in response to a positive video than to a neutral or a negative video [27]. Even when watching negative emotional videos (i.e. drama), increases in positive affect, but not in negative affect, predicted increases in group identification [26]. These results highlight the importance of shared attention in emotional contexts for fostering social bonding, although they are contradictory regarding whether this applies similarly to positively valenced and negatively valenced emotional experiences.

On the other hand, previous studies revealed that social bonding varied with the similarity (convergence and synchronization) of emotions in the group [28]. Participants jointly attending emotional videos exhibited convergent and synchronized emotional responses as measured by facial expressions [29–31] and autonomic physiological signals [30–33]. Notably, studies reported higher facial, cardiac and electrodermal synchrony between participants who were watching emotional videos seated next to each other than between participants who did not take part in the experiment together [30–33]. In other words, synchrony resulted from the alignment of responses between co-present participants attending the same stimulus together and not only from participants being exposed to the same stimulus. Crucially, the synchrony of positive facial expressions and electrodermal activity predicted the extent to which participants felt connected to each other [30,31]. For example, Cheong and collaborators [31] analysed the relations between synchrony and self-reported connectedness in dyads of unacquainted participants watching episodes of a TV show. The authors found that average ratings of connectedness correlated both with facial synchrony of positive emotion expressions and with electrodermal synchrony related to negative arousing emotions. These studies are of pivotal importance to clarify the relation between social bonding and emotional similarity due to non-verbal communication (i.e. facial expressions), but they leave open some questions.

In particular, it is unclear whether social bonding requires that individuals experience intense emotions, similar emotions or both, and whether these effects depend on joint attention and interpersonal alignment. Moreover, it is unclear whether these effects generalize across positively and negatively valenced emotional contexts. On the one hand, social bonding may increase as a function of emotional similarity due to interpersonal alignment. On this view, social bonding would increase in the context of joint attention, but not when individuals experience similar emotions in isolation, without being able to perceive and influence the emotions of other individuals. On the other hand, social bonding may increase as a function of emotional intensity, even in the absence of interpersonal alignment.

The current research aimed at investigating the effects of emotional intensity and emotional similarity (convergence and synchrony) on social bonding, as well as their interactions with the effects of joint attention and emotional valence. To this end, we measured subjective experiences, physiological activity and social attitudes in dyads of unacquainted individuals attending emotional videos together. We manipulated the emotional valence of the videos (positive, negative, neutral) and the type of shared attention between participants (joint, disjoint). Hence, we were able to test for the contribution of interpersonal alignment during the experiment and simultaneously to evaluate whether previous findings generalize to both negatively and positively valenced emotional contexts. Crucially, we transformed individual measures into group-level indices of emotional synchrony (physiology), emotional convergence (subjective experience) and social bonding (feeling of connectedness, social identification and desire for future interactions). Previous studies have typically approximated social bonding as the average of social attitudes within the group. Yet, since we believe that social bonding requires reciprocity in social attitudes, we took reciprocity within dyads into account. We expected that the measures of emotional intensity, emotional similarity and social bonding would increase with joint attention and the induction of positive and negative emotions.

We formulated two main hypotheses based on previous studies. First, we hypothesized that social bonding would increase in the context of joint attention as a function of emotional similarity (e.g. [30,31]). Second, we hypothesized that social bonding would increase in the context of joint attention as a function of emotional intensity (e.g. [25,26]). Given the lack of previous research, we had no specific expectation regarding whether the effects of emotional similarity and intensity are interdependent or additive.

## 2. Material and methods

### 2.1. Participants

We calculated *a priori* (*ɑ* = 0.05, power = 0.80) the minimum sample size needed to replicate the effect of co-presence on cardiac synchrony during emotion-eliciting videos [32]. In the context of the negative video, replicating this effect required 56 dyads. To anticipate potential technical problems, we thus recruited 122 French individuals randomly paired in 61 same-sex dyads of unacquainted individuals. The list of inclusion criteria included being 18- to 35-year-old, not suffering from auditory or visual impairment and reporting no history of neurological or psychiatric disorders. Two dyads were excluded due to lack of audio feedback during stimulus presentation, two dyads due to failure in the synchronization of the stimuli markers and one dyad due to displacement of electrocardiogram (ECG) electrodes during the experiment. The final sample (*n* = 112 individuals, 56 females) had a mean age of 23.48 ± 4.76 years. We used stratified randomization [34] to assign dyads within each sex group (two levels: male, female) to one of the two equally sized experimental groups (two levels: disjoint attention, joint attention) and, then, to one of the possible sequences of emotional stimuli (nine levels). The participants provided informed written consent and received monetary compensation (30€) for their participation.

### 2.2. Experimental procedure

**Figure 1.**
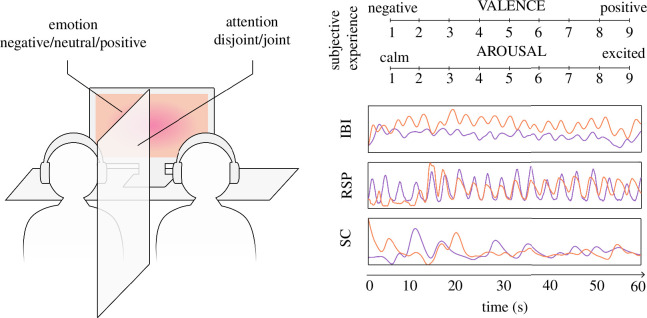
Schematic representation of the experimental setup and individual measures of emotion with examples of real physiological data. Time series represent pre-processed standardized physiological activity during the first minute of the negative video. Note that valence and arousal were measured with the pictorial scales of the SAM [35] that are not reproduced here. IBI, cardiac inter-beat interval; RSP, respiration (standardized tidal volume); SC, skin conductance (standardized phasic activity).

Before coming to the laboratory, individuals filled an online questionnaire assessing trait anxiety. On the day of the experiment, participants of the same dyad were welcomed separately. They read and signed an informed consent form and received general instructions, which included avoiding communication with the other participant throughout the experiment and refraining from moving during stimulus presentation. In compliance with sanitary guidelines concerning the COVID-19 pandemics, participants had to wear sanitary face masks throughout the experiment. They were equipped with physiological sensors and an audio headset and seated side by side in a dimly lit sound-attenuated room in front of an LCD monitor (24-inch diagonal, 60 Hz), as illustrated in figure 1. The distance between participants was 60 cm, and the distance from eye to screen was approximately 1 m. For dyads assigned to disjoint attention, an opaque curtain was drawn between participants to prevent non-verbal communication and interpersonal alignment between participants [29]. Finally, the experimenters left the room for the duration of the experiment.

We designed the task so that participants would take turns in answering questionnaires in a random fashion. Participants were instructed to follow written instructions and auditory cues indicating which participant had to answer the questions (e.g. ‘participant seating on the left’), and when to close their eyes to remain blind to the answers of the other participant. At the beginning of the experiment (*t*_0_), each participant was asked whether they knew the other participant. They had to report their current level of anxiety and their attitudes towards the other participant (social identification and desire for future interactions). To achieve emotional and physiological baseline, participants were then asked to relax for 5 min, while a fixation cross was displayed on-screen. They watched three 5-min emotion-eliciting videos in a random order, counterbalanced across the two groups (joint, disjoint). After each video, participants reported their subjective experiences of emotion and connectedness, followed by a fixed 2-min break. After watching all three videos (*t*_1_), participants again reported their attitudes towards the other participant as well as their own demographics (sex, age, education in years). Finally, they were debriefed and compensated. The approximate total duration of the experiment was 1 h 30 min. The code for running the experiment was written on MATLAB R2019b [36] using Psychtoolbox 3.0 [37,38] and CogToolbox [39].

### 2.3. Stimuli

Three videos were manually edited and subsequently validated in an online study (*n* = 50 participants; see electronic supplementary material). The positive video consisted of excerpts of the French comedy ‘The Intouchables’ [40]. The negative video consisted of excerpts of the documentary ‘Earthlings’ [41] depicting the sufferings of captive animals. The neutral video consisted of footage of a university library found on YouTube (FreeHD videos – [42]). The three excerpts were 5-min long and did not require prior knowledge about the story of the films to be understood. The online validation study confirmed that the videos reliably induced, respectively, positive valence (i.e. amusement), negative valence (i.e. sadness) and neutral valence for the control video.

### 2.4. Self-reports

Trait and state anxiety were measured using the French version of the State-Trait Anxiety Inventory test (STAI-T Y-1, STAI-T Y-2 [43,44]), to exclude individuals with high anxiety. At *t*_0_ and *t*_1_, participants rated on 7-point Likert scales their desire for future interactions with their co-participant ([45]; e.g. ‘Would you like to meet this person?’) and their degree of identification to their co-participant (We-scale [46]; ‘To what extent would you use the term “we” to describe your relationship with this person?’).

After each video, participants reported whether they had seen the video before (binary choice) and rated their experience of emotional arousal and valence during the video on the 9-point version of the Self-Assessment Manikin [35]. They also filled the Collective Effervescence Scale [7] assessing on 7-point Likert scales the feelings of connectedness (e.g. ‘I felt as if most everyone there felt the same emotions’) and sacredness elicited by the event (e.g. ‘I felt as if there was something special about the event’). Given our main hypotheses, we did not include feelings of sacredness in the analysis. The responses on multi-item scales were averaged, yielding reliable indices of desire for future interactions at *t*_0_ and *t*_1_ (seven items, *α* > 0.81) and feeling of connectedness after each video (four items, *α* > 0.77).

Thus, we measured three facets of social bonding: the feeling of connectedness during the shared experience, the change in social identification and the change in desire for future interactions. These measures correspond to theoretically distinct constructs: the feeling of connectedness is a measure of one aspect of collective effervescence (the subjective experience), whereas social identification and desire for future interactions are measures of cognitive changes related to self-categorization and motivation that can precede and follow from experiencing connectedness. Previous theoretical and empirical research highlighted that these phenomena are related but distinct [2,8,47].

### 2.5. Physiological measures

We continuously recorded ECG, respiratory activity (RSP) and skin conductance (SC) during the experiment. The corresponding signals are complementary non-invasive measures of autonomic arousal [48,49]. SC primarily reflects the influence of the sympathetic branch of the autonomic nervous system [50], whereas cardiac activity reflects the influence of both the parasympathetic and sympathetic branches of the autonomic nervous system and interacts with RSP [51]. Therefore, these three physiological measures can provide complementary information about emotion similarity and its relation to social bonding. The physiological signals were simultaneously sampled at 1 kHz and visualized using dedicated equipment and software (ADInstruments, Amsterdam, NL, USA). Data pre-processing was carried out on the three epochs of interest (i.e. videos) using the Fieldtrip toolbox [52] implemented on MATLAB R2019b [36].

ECG was recorded using disposable AgCl electrodes in Lead II configuration and amplified prior to digitization (Dual BioAmp, ADInstruments). Offline, the signal was band-pass filtered between 1 and 100 Hz (Butterworth, 4th order). For each video, a template QRS complex was computed and convolved with the raw ECG. R-peaks were automatically detected as peaks in the normalized convoluted signal exceeding 0.6 standard deviation, and the ECG was visually inspected to correct for peak misidentification. Inter-beat interval (IBI) was defined as the time distance in milliseconds (ms) between consecutive R-peaks. We computed the mean IBI as an inverse index of heart rate for each participant and video. We interpolated between consecutive IBI values (cubic spline, resampling at 20 Hz) to produce a smoothed time series. We then band-pass filtered the IBI time series between 0.04 and 0.4 Hz (Butterworth, 4th order) to focus on the standard low- and high-frequency (LF and HF) bands of the heart rate variability spectrum.

RSP was recorded in microvolts (mcV) using a thoracic respiration transducer belt, which measured changes in the circumference of the participant’s chest as a proxy for tidal volume. Offline, local peaks were automatically detected to identify expiration onsets, and the resulting signal was visually inspected to correct for peak misidentification. We computed the mean number of expiration onsets per minute as an index of respiratory rate.

SC was recorded in microsiemens (μS) using a pair of bipolar steel finger electrodes placed on the middle phalanxes of the index and middle fingers. After installation of the electrodes, participants were instructed to keep the hand in a still and relaxed position. As implemented in LabChart, before starting the recording, the participant’s absolute level of SC at that time was automatically subtracted from the subsequent recording (subject-zeroing) to account for interpersonal variability in baseline levels of SC. The signal was amplified prior to digitization (Bio Amp, ADInstruments). Offline, we band-pass filtered the signal between 0.05 and 5 Hz (first order, Butterworth) to focus on the phasic component of the electrodermal response [50].

### 2.6. Statistical outliers and physiological artefacts

Statistical outliers were defined as data points smaller (resp. greater) than the first (resp. third) quartile minus (resp. plus) three times the interquartile range of the sample. Our data set did not include any outlier on the scales of trait and state anxiety. Physiological artefacts were defined for each autonomic measure. For ECG, there was no artefact that could have impeded R-peak identification: *N*_ECG_ = 112 individuals. For RSP, we rejected participants when the maximum number of consecutive null values exceeded 5% of the duration of the entire video (i.e. constant respiratory signal due to a loosely attached belt), or when the variance of the signal exceeded three times the standard deviation above the sample mean (i.e. noisy data due to belt dysfunction) for at least one of the recordings: *N*_RSP_ = 105 participants. For SC, we rejected participants when the number of electrodermal responses was null for all three videos (i.e. unresponsive participants), or when implausible slope values (manually labelled or exceeding 2 μS in absolute value [53]) exceeded 5% of the duration of the entire video for at least one of the recordings: *N*_EDA_ = 99 participants. After the exclusion of dyads with artefacts, the sample size for testing our main hypotheses was *n* = 39 dyads.

### 2.7. Dyad-level indices

All of the measures were treated separately: for each type of measure, individual responses were aggregated into separate indices. Mathematical equations for data treatment are available in the electronic supplementary material and described below. For subjective emotional experience (valence, arousal), individual ratings were aggregated at dyad level into two types of indices. First, for each measure of subjective emotion and each video, we averaged individual ratings within each dyad to obtain two indices of emotional intensity: dyadic mean valence and dyadic mean arousal. We applied the same treatment to physiological measures to obtain three dyad-level physiological indices for each video: dyadic mean IBI, dyadic mean respiratory rate and dyadic mean SC level. Second, for each measure of subjective emotion and each video, we computed the absolute value of the difference between individual ratings within the dyad. As a consequence, we obtained two indices of emotional dissimilarity within the dyad: dyadic difference of valence and dyadic difference of arousal.

In addition, measures of social bonding were transformed to reflect both the intensity and the reciprocity of prosocial attitudes of dyad members. We computed the product of individual ratings within the dyad to obtain an index of mutual connectedness for each video. Therefore, the higher and the more similar the feelings of connectedness of dyad members, the greater the index of mutual connectedness. Like connectedness, social identification and desire for future interactions measured at the beginning and at the end of the experiment were separately transformed into dyadic products corresponding to mutual identification and mutual desire for interactions indices, respectively. Then, for these two measures separately, we subtracted the dyadic product at *t*_0_ from the dyadic product at *t*_1_. As a result, we obtained two indices reflecting, respectively, the change in mutual social identification and the change in mutual desire for interactions within each dyad. In other words, mutual social identification and mutual desire for future interactions reflected whether the intensity and reciprocity of prosocial attitudes within each dyad increased (positive value) or decreased (negative value) over the course of the experiment.

**Figure 2.**
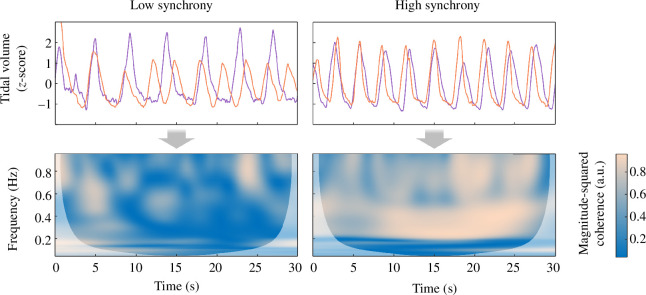
Co-participants' individual time series of repiratory activity over 30 s during the negative video (top) and the corresponding dyadic WTC as a function of time and frequency (bottom) for two dyads showing low synchrony (left) and high synchrony (right). Darker blue areas represent low synchrony and lighter orange areas represent high synchrony. The areas in lighter white shades represent the cones of influence, which are excluded from the computation of average synchrony. Magnitude-squared coherence is expressed in arbitrary units (a.u.).

For physiological synchrony, we computed the wavelet transform coherence (WTC). WTC represents the absolute correlation coefficient between time series as a function of time and frequency, ranging from 0 (no synchronization) to 1 (perfect synchronization). We computed WTC by applying the procedure detailed in Grinsted *et al.* [54] and implemented in MATLAB (‘wcoherence’ function). In brief, we first applied continuous Morlet wavelet transform over logarithmic scales to each individual pre-processed physiological signal (cardiac IBI, tidal respiratory volume, phasic SC). For each type of physiological signal and each video, we then computed WTC between the signals of the two co-participants (see the electronic supplementary material for a full description and mathematical equations). Finally, we averaged WTC values across time and frequency [55] to obtain an index of synchrony within each dyad, for each physiological measure and for each video. Figure 2 illustrates individual physiological signals and the corresponding dyadic values of WTC in the case of low and high synchrony.

WTC has several advantages compared to correlational measures that assume the stationarity of the signals. Indeed, physiological time series are highly auto-correlated and non-stationary, which can lead to the incorrect assessment of the significance of correlation coefficients [56]. In comparison, WTC is applicable to different types of bio-behavioural time series [55,57,58]. Moreover, WTC enables capturing synchrony at different frequencies, which has the potential of distinguishing between types of synchrony related to distinct physiological mechanisms. The distinction between parasympathetic and sympathetic autonomic activity sometimes translates into different patterns of interpersonal physiological synchrony [55,59,60]. For example, Danyluck & Page-Gould [59] found that the social context (cooperation versus competition, verbal communication) modulated the relation of affiliation with parasympathetic synchrony but not with sympathetic synchrony. As such, WTC is particularly relevant to isolate parasympathetic synchrony from sympathetic synchrony in the case of ECG. Consequently, for ECG, we averaged WTC values separately on the standard LF (0.05–0.15 Hz) and HF (0.15–0.4 Hz) bands of the heart rate variability spectrum, because they reflect the influence of distinct physiological mechanisms [61]. Finally, we assessed the bivariate correlations between WTC and other methods for computing synchrony (e.g. Pearson’s correlations, cross-recurrence quantification metrics), and we replicated our findings with correlational measures (see the electronic supplementary material).

### 2.8. Statistical analysis: regressions and post hoc tests

Statistical modelling was performed using R (v. 4.2.1; [62]) and RStudio [63]. All models were based on linear regressions with the identity link function (‘lme4’ package, [64]), except for ordinal logistic regressions with the cumulative logit link function (‘ordinal’ package, [65]) to model discrete ratings of arousal and valence.

As a general approach, continuous predictors were centred around the mean of the experimental sample and scaled (*z*-scored), which enhances the interpretability of the regression coefficients in models involving interaction terms [66]. Categorical predictors were coded as treatment dummy variables, so that lower-level predictors reflected simple effects. Models were fitted based on restricted maximum likelihood unless otherwise stated, when maximum likelihood was used to compare between models with different fixed effects [67]. Degrees of freedom and *p* values were approximated from likelihood-ratio tests (LRTs; [68]) by using the Welch–Satterthwaite equation. We used the coefficient of determination (*R* ^2^) to assess the goodness-of-fit of our models, and we compared between alternative models based on their log-likelihood. In case of a significant interaction between predictors, we estimated the conditional effects at factor levels and performed pairwise comparisons (‘emmeans’ package, [69]). Our experimental design involved within-participant and within-dyad measures: individual responses for each video were nested within participants, and participants were themselves nested within dyads. Therefore, we ran mixed-effect regressions including random effects to account for statistical non-independence of within-dyad data. Specifically, we modelled random intercepts for dyads, crossed with video valence (negative, neutral, positive) and video index (first, second, third). When applicable, we included all repeated-measure predictors as random slopes within the levels of dyads, crossed with video valence and video index [70].

For frequentist post hoc tests, we evaluated data normality with Shapiro–Wilk tests [71]. Given non-normal distributions of variables (valence, arousal, connectedness, physiology), we opted for non-parametric tests. Specifically, we computed pairwise Spearman’s correlation coefficients [72] between variables, and we evaluated the differences between groups (joint, disjoint) with two-tailed Wilcoxon–Mann–Whitney tests [73]. Effect sizes were reported as Pearson’s coefficients, and *p* values for post hoc comparisons were adjusted with Holm’s method [74] to correct for family-wise error rate.

### 2.9. Statistical analysis: manipulation check

As a pre-requisite for testing our main hypotheses, we assessed the effects of our experimental manipulations: we ran mixed-effects regressions of dyad-level indices of emotional intensity, emotional similarity and social bonding. For emotional intensity, we conducted ordinal logistic mixed-effect regressions of emotional valence and emotional arousal, and we conducted linear mixed-effect regressions of cardiac IBI, respiration rate and SC, in which the dependent variables represented the mean of individual values within the dyad. For emotional similarity approximated as the convergence of subjective reports, we conducted ordinal logistic mixed-effect regressions of dyadic difference of emotional valence and dyadic difference of emotional arousal. For emotional similarity approximated as the synchronization of physiology, we conducted linear mixed-effect regressions of synchrony in cardiac synchrony, respiratory synchrony and electrodermal synchrony. For social bonding, we conducted a linear mixed-effect regression of mutual connectedness.

All of these regressions estimated the fixed effects of video valence (negative versus neutral, positive versus neutral), attention type (joint versus disjoint), their interaction and the following covariates: previous exposure to the video (both participants versus mixed exposure, none of the participants versus mixed exposure), sex (female versus male), demographic variables (age, education) and anxiety (trait and state). These models also included random intercepts for dyads crossed with video index to account for the effect of time. We then ran similar regression analyses excluding responses to the neutral video to directly compare responses to the positive and negative videos (positive versus negative).

### 2.10. Statistical analysis: main hypotheses

To test our two main hypotheses, we modelled mutual connectedness in response to the positive and negative videos following a two-step procedure. First, we devised a full model estimating the fixed effects of the dyad-level variables of emotional intensity and emotional similarity, attention type (joint versus disjoint) and their interaction terms. Crucially, we excluded from the full model the variables that were not manipulated by the experimental design, hence, that we could not confidently interpret as reflecting interpersonal emotional intensity and emotional similarity. We included the following covariates: baseline measures of social bonding (mutual social identification at *t*_0_, mutual desire for interaction at *t*_0_), their respective interaction with attention type and demographic variables (sex, age, education). The random structure of the complete model was composed of random intercepts and slopes for indices of emotional intensity and similarity within the levels of the dyads crossed with video valence and video index. Prior to modelling, we corrected the dependent and independent variables by subtracting the values corresponding to the neutral video from the values corresponding to, respectively, the positive and negative videos. In doing so, we assessed the relations between changes in the independent variables and the dependent variable (mutual connectedness) in valenced emotional contexts as compared to a neutral baseline.

Second, we implemented an automatic selection procedure designed to increase statistical power and to avoid inflating Type I error rate (‘buildmer’ package, [75]). The procedure started from the complete model specified at the previous step [70] and ordered the fixed effects as a function of their contribution to the significance of the change in LRT. If the full model was too complex to converge, the effects that contributed the least were excluded until the model could converge. Then, to avoid overfitting, fixed effects were discarded one by one from this model based on the LRT (backward stepwise elimination; [76]), until all fixed effects in the model were significant. This procedure ensured that the most parsimonious set of statistical predictors were retained in the model. As part of this procedure, models were fitted based on maximum likelihood so that we could compare between models with different fixed effects. We compared the final model resulting from this procedure with models resulting from alternative procedures (i.e. forward stepwise elimination, full model without a selection procedure). These comparisons are reported in the electronic supplementary material.

Post hoc, we also assessed the bivariate correlations between mutual connectedness and, respectively, mutual social identification and mutual desire for future interactions as a function of video valence (positive versus neutral, negative versus neutral) and attention type (joint, disjoint). These post hoc tests evaluated whether mutual feelings of connectedness during shared experiences related to other facets of social bonding, measured as changes in social attitudes between co-participants over the course of the experiment.

## 3. Results

### 3.1. Manipulation check

**Figure 3.**
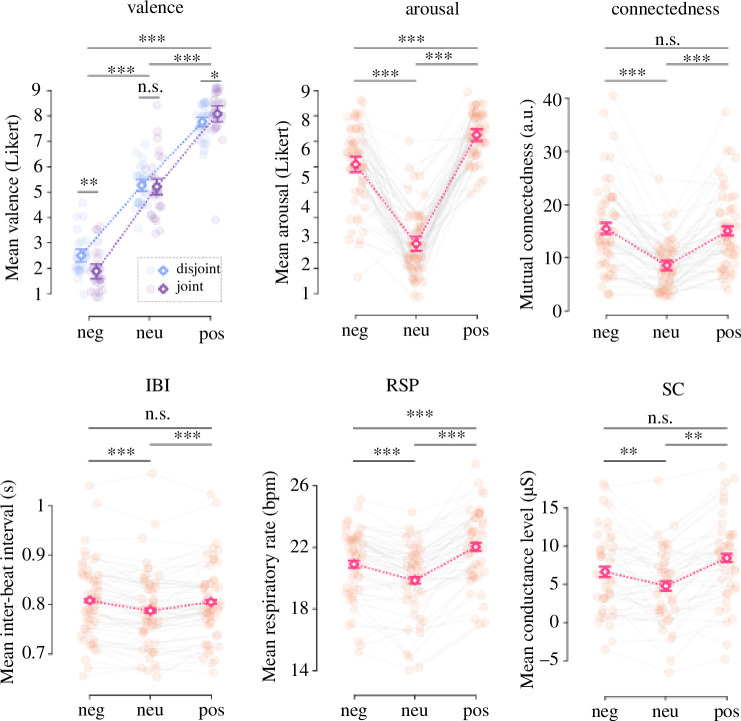
Effects of the experimental manipulation on dyad-level indices of emotional intensity and mutual connectedness. Dyad-level indices of emotional intensity are divided into subjective emotion (valence, arousal) and physiology (IBI, RSP, SC). SC values have been baseline corrected at individual level prior to averaging individual values within the dyad. Points represent dyad-level data with a random vertical and horizontal jitter for visualization purposes; white diamonds represent mean values; error bars represent 95% confidence intervals around the mean after correction for within-dyad variability. Stars indicate the results of pairwise comparisons between estimated marginal means. *p < 0.05; **p < 0.01; ***p < 0.001. IBI, inter-beat interval; RSP, respiration; SC, skin conductance.

There was no significant difference in age, education, anxiety (trait, state) and affiliative attitudes at *t*_0_ (social identification, desire for future interactions) between the two groups assigned to joint and disjoint attention, respectively (*p* > 0.24). We analysed the effects of our experimental design (valence of the video, type of attention) on dyad-level indices of emotional intensity, emotional similarity and mutual connectedness. Table 1 provides summary statistics of dyad-level variables, and figure 3 illustrates the results. Please refer to the electronic supplementary material for the full regression tables.

**Table 1.** Summary statistics for dyad-level variables as a function of video valence.

parameter	*N_dyads_*	negative video			neutral video			positive video		
		M	s.d.	Mdn	M	s.d.	Mdn	M	s.d.	Mdn
mean valence (Likert)	56	2.20	0.82	2.00	5.23	1.02	5.00	7.94	0.86	8.00
mean arousal (Likert)	56	6.09	1.59	6.50	2.96	1.28	2.50	7.25	0.94	7.50
mean IBI (ms)	56	808	76.00	808	788	77.3	787	805	76.00	797
mean RSP rate (expirations per minute)	49	20.90	2.34	21.70	19.90	2.39	20.50	22.00	2.34	22.10
mean SC level (μS)	44	6.69	5.30	6.30	4.81	4.84	4.92	8.48	5.56	8.81
difference valence (Likert)	56	1.25	1.16	1	1.79	1.58	1	1.30	1.11	1
difference arousal (Likert)	56	2.32	1.72	2	2.18	1.87	1.5	1.89	1.81	1
synchrony IBI LF (a.u.)	56	0.35	0.04	0.34	0.34	0.05	0.34	0.36	0.05	0.35
synchrony IBI HF (a.u.)	56	0.36	0.07	0.35	0.35	0.07	0.34	0.35	0.07	0.36
synchrony RSP (a.u.)	49	0.34	0.06	0.34	0.34	0.05	0.34	0.35	0.06	0.35
synchrony SC (a.u.)	44	0.32	0.05	0.33	0.32	0.05	0.32	0.33	0.05	0.34
mean connectedness (a.u.)	56	3.90	0.99	3.88	2.98	0.73	3.06	3.90	0.85	3.94
mutual connectedness (a.u.)	56	15.5	8.25	13.7	8.56	4.50	8.22	15.1	7.20	14

HF, high-frequency; IBI, inter-beat interval; LF, low-frequency; M, mean; Mdn, median; RSP, respiration; SC, skin conductance; s.d., standard deviation.

Regarding emotional intensity indexed by the average ratings of subjective emotion within each dyad, the negative video was more likely than the neutral video to elicit negative valence [Odds Ratio = 0.05, 95% CI (0.02, 0.10), *p* < 0.001] and the positive video was more likely than the neutral video to elicit positive valence [*OR* = 12.35, 95% CI (5.64, 27.05), *p* < 0.001]. Dyads were also more likely to report high arousal in response to the negative video [*OR* = 6.25, 95% CI (3.40, 11.49), *p* < 0.001] and the positive video [*OR* = 16.59, 95% CI (7.85, 35.07), *p* < 0.001] than in response to the neutral video. For subjective valence, we found a significant interaction between attention type and video valence [positive: *OR* = 2.21, 95% CI (1.02, 4.77), *p* = 0.04; negative: *OR* = 0.43, 95% CI (0.20, 0.95), *p* = 0.04]. In comparison to disjoint dyads, joint dyads were more likely to report negative valence in response to the negative video (*OR* = 0.46, *SE* = 0.15, *p* < 0.01) and positive valence in response to the positive video (*OR* = 0.35, *SE* = 0.14, *p* = 0.03). Furthermore, the positive video was more likely to elicit positive valence [*OR* = 71.73, 95% CI (18.94, 271.71), *p* < 0.001] and high arousal [*OR* = 2.67, 95% CI (1.68, 4.24), *p* < 0.001] than the negative video.

Regarding emotional intensity indexed by the average of physiological responses within each dyad, the positive and negative videos elicited longer mean cardiac IBI [positive: *β* = 24.44, 95% CI (13.89, 34.98), *p* < 0.001; negative: *β* = 18.84, 95% CI (10.70, 26.98), *p* < 0.001], higher mean respiratory rate [positive: *β* = 1.95, 95% CI (1.15, 2.75), *p* < 0.001; negative: *β* = 1.00, 95% CI (0.40, 1.59), *p* < 0.01] and higher skin conductance level [positive: *β* = 2.52, 95% CI (0.56, 4.48), *p* = 0.01; negative: *β* = 1.68, 95% CI (0.14, 3.22), *p* = 0.03] than the neutral video. The positive video elicited higher respiratory rate [*β* = 1.04, 95% CI (0.16, 1.93), *p* = 0.02] than the negative video.

Regarding emotional similarity indexed by the difference in subjective emotion and the synchrony of physiological activity within each dyad, we did not find systematic effects of video valence, attention type or their interaction. We only observed marginal effects of the positive video, which elicited higher cardiac LF synchrony [*β* = 0.02, 95% CI (0.00, 0.04), *p* = 0.08], higher respiratory synchrony [*β* = 0.02, 95% CI (0.00, 0.05), *p* = 0.09] and higher electrodermal synchrony [*β* = 0.02, 95% CI (0.00, 0.05), *p* = 0.10] than the neutral video. Moreover, a non-parametric bootstrapping procedure showed no difference in emotional similarity in the context of joint attention between experimental dyads composed of co-present participants and surrogate dyads composed of participants who did not participate in the experiment together (see electronic supplementary material for the methods and results). Post hoc, we checked whether dyadic emotional convergence and synchronization could instead reflect a byproduct of emotional intensity induced by the positive and negative videos. We found a significant correlation between difference in arousal and mean arousal: *ρ* = −0.36, *p*_Holm_ < 0.01.

Regarding social bonding indexed by mutual connectedness, we found significant effects of video valence [positive: *β* = 5.39, 95% CI (2.35, 8.42), *p* = 0.001; negative: *β* = 6.27, 95% CI (3.89, 8.65), *p* < 0.001]. The difference in mutual connectedness between the positive and negative videos was not statistically significant: *β* = −0.46, 95% CI (−3.86, 2.93), *p* = 0.79.

### 3.2. Main hypotheses: emotion and social bonding

To test our main hypotheses, we modelled mutual connectedness in response to the positive and negative videos. Because dyad-level indices of emotional intensity, and not emotional similarity, were modulated by video valence and attention type, we only included emotional intensity and the covariates in the full model, before proceeding to model selection. Variables in the model reflected changes from the neutral video.

**Figure 4.**
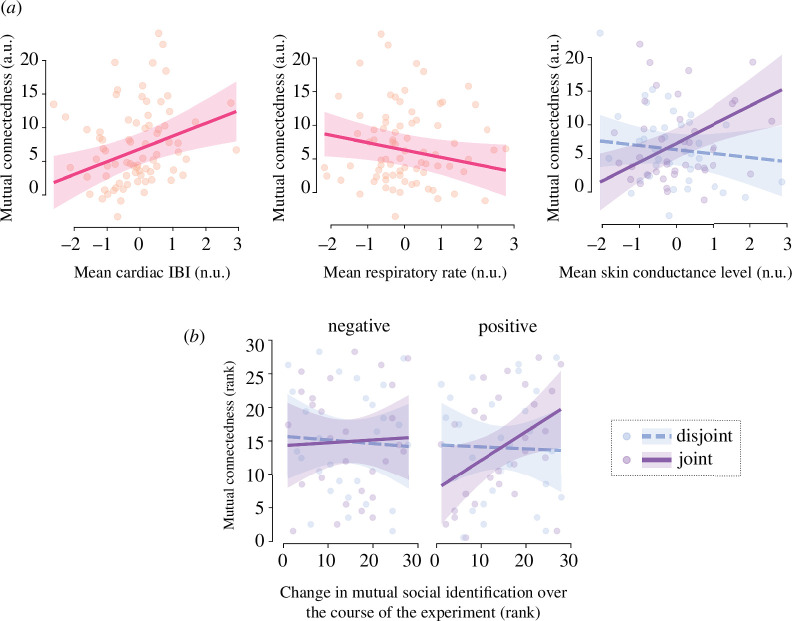
(*a*) Significant fixed effects in the final selected model of mutual connectedness (changes from the neutral video to the positive and negative videos); (*b*) bivariate correlations between mutual connectedness (changes from the neutral video to the positive and negative videos) and mutual social identification (changes from the start to the end of the experiment). Points represent dyad-level data; lines represent fitted linear predictions; shaded areas represent 95% confidence intervals. Mutual connectedness is expressed in arbitrary units (a.u.) and physiological variables in normalized units (n.u.).

As can be seen from table 2, the final selected model included significant effects of mean respiratory rate [*β* = −1.09, SE = 0.54, 95% CI (−2.18, 0.00), *p* = 0.05], mean cardiac IBI [*β* = 1.90, SE = 0.62, 95% CI (0.65, 3.14), *p* < 0.01] and a significant interaction between mean skin conductance level and attention type [*β* = 3.40, SE = 1.15, 95% CI (1.11, 5.70), *p* < 0.01]. An increase in mean skin conductance level predicted an increase in mutual connectedness in the context of joint attention [*β* = 2.78, SE = 0.90, 95% CI (0.98, 4.58)] but not in the context of disjoint attention [*β* = −0.62, SE = 0.84, 95% CI (−2.30, 1.06)]. Figure 4*a* illustrates the significant fixed effects in the model. A post hoc comparison between the final selected model and a nesting model additionally including indices of dyadic physiological synchrony showed no significant improvement in log-likelihood: *χ²*_(4)_ = 5.82, *p* = 0.21. We then ran similar automatic procedures to model mutual connectedness in response to the positive videos versus negative videos, respectively (see the electronic supplementary material).

**Table 2.** Final selected model of changes in mutual connectedness from the neutral video to the positive and negative videos (*n* = 78 observations).

statistical predictor	standardized estimate	standard error	95% CI lower limit	95% CI upper limit	***p* value**
intercept	6.56	1.18	4.14	8.97	**<0**.**001**
mean SC level × attention type	3.40	1.15	1.11	5.70	**<0**.**01**
mean IBI	1.90	0.62	0.65	3.14	**<0**.**01**
mean RSP rate	−1.09	0.54	−2.18	0.00	**0.05**
attention type (joint versus disjoint)	0.90	1.65	−2.47	4.27	0.59
mean skin conductance level	−0.62	0.80	−2.22	0.97	0.44
**random effects**					
number of clusters (*N*_dyads_)	39				
residual variance (*σ* ^2^)	10.77				
random-intercept variance (*τ*_00_)	20.97				
ICC	0.66				
**model performance**					
marginal *R* ^2^/conditional *R* ^2^	0.19/0.73				

CI, confidence interval; IBI, inter-beat interval; ICC, intra-class correlation; RSP, respiration; SC, skin conductance.

As illustrated in figure 4*b*, post hoc bivariate correlations corrected for multiple comparisons showed that mutual connectedness in response to the positive video, but not to the negative video, was marginally correlated with changes in mutual social identification, but only for dyads in joint attention: *ρ* = 0.42, *p*_Holm_ = 0.10. The correlation between mutual connectedness in response to the positive video and mutual desire for future interactions for dyads in joint attention was non-significant after correction for multiple comparisons: *ρ* = 0.32, *p*_Holm_ = 0.39. The full correlation tables are available in the electronic supplementary material.

## 4. Discussion

We explored the relationship between emotion and social bonding during shared experiences. In particular, we evaluated the effects of emotional intensity and joint attention on mutual prosocial attitudes within dyads. Overall, we found that joint attention and emotional intensity, indexed by dyadic physiological arousal, predicted social bonding between unacquainted individuals and that interpersonal alignment of emotions was not necessary for shared experiences to promote social bonding.

The results indicated that we successfully manipulated the mean intensity of emotional responses within dyads. The positive and negative videos were opposite to one another for subjective valence, and both were more subjectively and physiologically arousing than the neutral video. In particular, we found changes in heart rate, respiratory rate and skin conductance level that are congruent with previous reports of physiological responses to emotionally arousing stimuli [77,78]. Mean subjective valence within dyads also varied as a function of the attention manipulation: valence in response to the negative (resp. positive) video was more negative (resp. more positive) for joint attention than disjoint attention. This result matches previous evidence of the amplification of emotional experiences in the context of joint attention, which is due to increased allocation of cognitive resources to the processing of the stimulus [79]. In the present task, this result suggested that shared attention supporting social influence on emotion was successfully manipulated.

Regarding our first hypothesis, we did not find significant effects of the experimental manipulation on emotional similarity, indexed by the convergence of subjective experiences and the synchrony of physiological responses. Hence, contrary to our expectations, we deduced that the observed levels of convergence and synchrony were due neither to the induction of emotions nor to interpersonal alignment. We note a few specificities of the present study that could explain the difference with previous studies showing interpersonal alignment of emotions between participants jointly watching emotional videos [30–32]. For one, our experimental setup deliberately aimed at removing some of the channels of social influence between dyad members: even when they jointly attended, participants were explicitly instructed not to communicate, and they were wearing audio headsets. Moreover, given the COVID-19 pandemics at the time of data collection, participants were wearing face masks throughout the experiment, which may have diminished the perception of facial expressions of emotion [80]. Given that the processing of facial expressions supports emotional convergence [81] and physiological synchrony [82], the limited facial feedback is a plausible explanation of the absence of emotional alignment in our experiment. Besides, dyads were composed of unacquainted individuals, and previous research showed that common group membership and prior affiliation facilitate the convergence of emotions [83,84] and influence physiological synchrony [10,33]. In our study, unacquainted participants may have spontaneously avoided gazing at each other: we gave participants no explicit incentive to look at the other participant during the task, and gazing at strangers may have been perceived as a violation of social norms [85]. This said, as noted above, joint attention influenced subjective valence, indicating that the manipulation of attention influenced dyadic measures of emotion, irrespective of reduced visual access to facial expressions.

Accordingly, changes in emotional similarity could not explain changes in social bonding in the context of the present study. Our results showed that interpersonal alignment and emotional similarity are not necessary for individuals to reciprocally feel like they emotionally align with each other. It is worth stressing that we do not rule out the possibility that interpersonal alignment and emotional similarity contribute to social bonding in other contexts. There is evidence that physiological synchrony correlates with prosocial attitudes in various contexts (e.g. [86–88]), including jointly attending emotional videos [30–32]. Our results could indicate that the relation between social bonding and physiological synchrony during shared emotional experiences requires that individuals communicate and co-regulate their emotions (see [15] for a discussion of the functions of affective synchrony). In addition, a meta-analysis found that the relationship between physiological synchrony and relationship outcomes was small [89], suggesting the existence of alternative explanations of social bonding. In this regard, the present study hints at possible mechanisms of social bonding that do not require the convergence and synchronization of emotions.

Regarding our second hypothesis, we found that social bonding increased as a function of changes in dyadic emotional intensity induced by the positive and negative videos, while taking potentially confounding factors into account, such as similarity in demographics (i.e. age) and first impressions (i.e. affiliative attitudes at *t*_0_). Previous studies showed that the effect of joint attention on prosocial attitudes depended on the appraisal of video clips [90] and on the emotion they elicited [25,26]. By comparing valenced emotional contexts with a neutral emotional baseline, the current study revealed that changes in mean physiological arousal within dyads (lower heart rate, lower respiratory rate and higher skin conductance level) predicted changes in social bonding (higher mutual connectedness). Furthermore, an increase in mutual connectedness in response to the positive video predicted an increase in mutual social identification over the course of the experiment for participants in joint attention. This finding supports the idea that feelings of connectedness reflect a fundamental drive to affiliate with other individuals [91]. It is also coherent with studies of collective effervescence, which link the subjective experience of positive emotional valence and emotional arousal to feelings of connectedness and to subsequent measures of group bonding (see [8] for a meta-analysis).

Research on collective effervescence so far mostly relied on self-reports of emotional intensity. In the present study, for the first time, we examined both the subjective and physiological components of emotional intensity, in relation to changes in mutual feeling of connectedness. Although subjective and physiological measures of emotion typically correlate with each other, previous work revealed that the strength of their correlation varies with individual and contextual differences, and that subjective and physiological measures of emotion have partially distinct brain correlates (for a review, see [92]). In the current study, it was the embodied component of emotional intensity, as measured by physiological arousal, more than the conscious experience of arousal, that was related to increased mutual feelings of connectedness in the context of joint attention. Previous studies showed relationships between physiological arousal and prosocial attitudes (e.g. [93]), notably in the context of social rituals (e.g. [10,94]). The effect of sympathetic activation on connectedness is also congruent with the description of imagistic rituals defined as low-frequency, small-scale and highly arousing rituals that promote reciprocal social identification by way of intense emotions [95]. Compared to previous research on collective effervescence and rituals, the present study indicated that the emergence of prosocial attitudes between strangers is associated with changes in physiology due to the induction of positive and negative emotions.

Moreover, we found that mean sympathetic activation within dyads (mean skin conductance level) predicted mutual feelings of connectedness for dyads in joint attention but not disjoint attention. A possible interpretation of this interaction stems from the assumption that conspecifics represent valuable resources to cope with the potentially detrimental physiological and psychological consequences of arousing emotional events (see [96] for a review of findings in the case of threat). Indeed, the positive relationship between dyadic sympathetic arousal and prosocial attitudes in the context of joint attention could reflect that participants were more inclined to entertain prosocial attitudes when their co-participants were visible and represented a potential source of social support. Granted, none of the videos used in this experiment, let alone the positive video, depicted acute threats. Nonetheless, previous research has shown that intense (positive or negative) emotional experiences motivate individuals to interact with others for diverse motives, including coping, clarification and bonding [97]. Our results suggested that joint attention is a prerequisite for this phenomenon in the context of shared experiences.

As a consequence, the present study sheds light on the relation between the presence of other individuals and the social outcomes of shared experiences. In theory, feelings of connectedness may arise independently of the presence of other individuals: ‘One can feel connected to someone else, without this person being necessarily “present” (real or virtual/mediated), or without necessarily being in interaction with him/her’ [98, p. 217]. In such contexts, prosocial attitudes may result from social projection, or the assumption that other individuals have mental states that are similar to ours [99]. This could explain that in our experiment, dyads always showed a level of connectedness superior to the minimal value, even when participants could not see each other (disjoint attention). However, the effect of joint attention in our study indicated that prosocial attitudes were also dependent on the presence of a visible co-attendee. Specifically, our manipulation of attention relied on a control group of participants who could not see each other, but knew they were watching the same video on a common screen and presumably experiencing similar emotions. Because participants were told they were watching the same videos, we manipulated one aspect of joint attention, namely perceptual awareness of a co-attending participant (e.g. [29]), whereas previous studies have sometimes manipulated both perceptual awareness of co-participants and the assumption that co-participants are watching similar or different videos (e.g. [25]). In our experiment, the observed effect of dyadic sympathetic activation on social bonding did not only depend on participants’ assumption that a co-attendee was present in the room (disjoint attention) but also on their perceptual awareness of the co-attendee (joint attention). Future research should investigate whether the same effects are observed when co-participants have visual access to one another but experience different events. Do prosocial attitudes emerge when co-participants are visible to each other but watch different videos or the same video asynchronously (one participant starting to watch the video before the other)? Moreover, as mentioned before, participants had limited access to the facial emotional expressions of their co-attendees. This suggests that although perceptual awareness of other individuals is a condition of the effect of dyadic sympathetic arousal, the direct perception of their emotions may not be. As described by the authors of a review of affiliation under threat: ‘affiliative behaviour increases as a function of the perception that the other person is facing the same situation rather than as a function of the perception that the other person is expressing the same emotional state’ [96, p. 302]. The limited communication of emotions in the present study also challenges Durkheim’s original conception of collective effervescence, which relied on overt emotional displays (for a detailed account, see [2]). Indeed, it has recently been proposed that collective effervescence can emerge in contexts that constrain the expression and perception of emotions, such as digitally mediated interactions [100,101].

The present study comes with specific limitations. First, we observed a significant difference in dyadic subjective arousal between the positive and negative videos (*r* = 0.55, *p*_Holm_ < 0.001). Although we did not observe such a difference during the pre-validation of the stimuli (see electronic supplementary material), and despite controlling for variations of slopes between the videos in our model of connectedness, we cannot exclude a possible overlap between the effects of inducing arousal and valence in our study. Future studies could alleviate this potential confound by more closely matching the levels of arousal elicited by pairs of positive and negative videos. Second, we chose to focus on the explanatory role of emotion in social bonding at the expense of other candidate factors. For example, we did not measure empathy-related traits, which are known to highly influence social bonding during joint activities [102,103]. Moreover, we left the measure of sacredness out of the analysis to focus on feelings of closeness, hence, capturing only part of the subjective experience of collective effervescence. Indeed, the subscales of connectedness and sacredness tap into conceptually distinct dimensions of collective effervescence [7]. Although both measures were significantly correlated across videos in our sample (*ρ* > 0.37, *p*_Holm_ < 0.001), previous work showed that self-transcendent feelings mediated the effect of interpersonal cardiac synchrony on identity fusion [86]. Third, previous research showed that watching negative films in a group induced changes in subjective pain threshold and positive affect, which independently predicted changes in identification to that group [26]. The authors interpreted the changes in pain threshold as evidence for the release of endorphins. This suggests that neuropeptides sustain social bonding during synchronized group activities [104]. Although we did not design our experiment to test this hypothesis, the activation of the endorphin system could partly explain the changes in social affiliation observed in the present study. Finally, the fact that participants were wearing face masks throughout the experiment may have reduced non-verbal facial communication and perception of facial emotional cues, which may limit the generalizability of our results to other types of shared experiences. We acknowledge that shared experiences represent a broad category in which the intensity and similarity of emotions may vary depending on the size of the group, the type of relationship between individuals and the communication of emotions. For participants in the joint condition, the shared experiences in our experiment resemble the shared experiences of unacquainted audience members who attend movies in theatre [105], where they sit side by side and usually refrain from addressing each other. This minimal type of shared experience differs in many ways from shared experiences involving communication and coordination such as singing, playing games or having a face-to-face conversation (e.g. [106–108]). Therefore, future research should assess the generalizability of the present results to shared experiences involving larger groups and repeated interactions.

## 5. Conclusion

To the best of our knowledge, the present study provides the first experimental demonstration that dyad-level emotional intensity indexed by physiological arousal predicts reciprocal prosocial attitudes in minimal social interactions, whether emotions are negative or positive, and in the absence of interpersonal emotional similarity between participants. In brief, we found that dyadic physiological arousal and joint attention predicted mutual feelings of connectedness, which correlated with changes in mutual social identification. Moreover, our results indicated that shared experiences need not involve interpersonal alignment of emotions to promote social bonding. Overall, we showed that social bonding between unacquainted individuals depends not only on who they are and how they feel about each other in the first place but also on the intensity of the emotions they experience and their perceptual awareness of the presence of each other.

Our results have several implications for our understanding of the social consequences of shared emotional experiences. First, we provided evidence refining the conceptual model of collective effervescence [2] and notably the role of physiological arousal. Second, our study showed a possible decoupling between objective measures of shared emotions (the convergence and synchronization of emotions) and the subjective experience of sharing (mutual feelings of connectedness), which suggests the existence of distinct and possibly additive mechanisms of social bonding during shared emotional experiences [14]. These results support the idea that shared emotional experiences represent a continuum that ranges from weakly to strongly shared emotions [11,109]. Strongly shared emotions involve the alignment of emotional responses between group members who explicitly communicate their emotions and who are mutually aware of experiencing similar emotions. In comparison, participants in our experiment presumably shared emotions in a weaker sense, which suggests that sharing emotions in a stronger, more collective sense is not a prerequisite for social bonding. Third, our results supported the hypothesis that emotion has a bonding function [110], as it could explain how brief one-shot interactions with strangers can contribute to satisfying the need to belong to social groups [111,112]. Finally, the current study could also explain why people seek out group activities that induce intense and arousing emotions, even participating in sad commemorations or attending dramatic narrative fictions that induce negatively valenced emotions [26,113].

## Data Availability

The data set and code supporting this article can be accessed on Dryad: [114]. Supplementary material is available online [115].

## References

[1] Durkheim E . 1915 The elementary forms of the religious life: a study in religious sociology. Swain JW, translator. London, UK: The Free press (Original work published 1912).

[2] Rimé B , Páez D . 2023 Why we gather: a new look, empirically documented, at Émile Durkheim’s theory of collective assemblies and collective effervescence. Perspect. Psychol. Sci. **18** , 1306–1330. (10.1177/17456916221146388) 36753611

[3] Castro-Abril P , Da Costa S , Navarro-Carrillo G , Caicedo-Moreno A , Gracia-Leiva M , Bouchat P , Cordero B , Méndez L , Páez D . 2021 Social identity, perceived emotional synchrony, creativity, social representations, and participation in social movements: the case of the 2019 Chilean Populist protests. Front. Psychol. **12** , 764434. (10.3389/fpsyg.2021.764434) 34955983 PMC8699020

[4] von Scheve C , Ismer S , Kozłowska M , Solms-Baruth C . 2017 Rituals, emotional entrainment and national identification: a cross-national study around the European football championship. Comp. Soc. **16** , 585–612. (10.1163/15691330-12341439)

[5] Liebst LS . 2019 Exploring the sources of collective effervescence: a multilevel study. Sociol. Sci. **6** , 27–42. (10.15195/v6.a2)

[6] Collins R . 2014 Interaction ritual chains and collective effervescence. In Collective emotions: perspectives from psychology, philosophy, and sociology (eds C von Scheve , M Salmela ), pp. 299–311. Oxford, UK: Oxford University Press. (10.1093/acprof:oso/9780199659180.003.0020)

[7] Gabriel S , Naidu E , Paravati E , Morrison CD , Gainey K . 2020 Creating the sacred from the profane: collective effervescence and everyday activities. J. Posit. Psychol. **15** , 129–154. (10.1080/17439760.2019.1689412)

[8] Pizarro JJ , Zumeta LN , Bouchat P , Włodarczyk A , Rimé B , Basabe N , Amutio A , Páez D . 2022 Emotional processes, collective behavior, and social movements: a meta-analytic review of collective effervescence outcomes during collective gatherings and demonstrations. Front. Psychol. **13** , 974683. (10.3389/fpsyg.2022.974683) 36118463 PMC9473704

[9] Moors A . 2009 Theories of emotion causation: a review. Cogn. Emot. **23** , 625–662. (10.1080/02699930802645739)

[10] Konvalinka I , Xygalatas D , Bulbulia J , Schjødt U , Jegindø EM , Wallot S , Van Orden G , Roepstorff A . 2011 Synchronized arousal between performers and related spectators in a fire-walking ritual. Proc. Natl Acad. Sci. USA **108** , 8514–8519. (10.1073/pnas.1016955108) 21536887 PMC3100954

[11] Salmela M . 2012 Shared emotions. Philos. Explor. **15** , 33–46. (10.1080/13869795.2012.647355)

[12] Goldenberg A , Garcia D , Halperin E , Gross JJ . 2020 Collective Emotions. Curr. Dir. Psychol. Sci. **29** , 154–160. (10.1177/0963721420901574)

[13] von Scheve C , Salmela M . 2014 Collective emotions: perspectives from psychology, philosophy, and sociology. Oxford, UK: Oxford University Press. (10.1093/acprof:oso/9780199659180.001.0001)

[14] Chung V , Grèzes J , Pacherie E . 2024 Collective emotion: a framework for experimental research. Emot. Rev. **16** , 28–45. (10.1177/17540739231214533)

[15] Wood A , Lipson J , Zhao O , Niedenthal P . 2021 Forms and functions of affective synchrony. In Handbook of embodied psychology: thinking, feeling, and acting (eds M Robinson , L Thomas ), pp. 381–402. Cham, Switzerland: Springer. (10.1007/978-3-030-78471-3_17)

[16] Parkinson B . 2020 Intragroup emotion convergence: beyond contagion and social appraisal. Pers. Soc. Psychol. Rev. **24** , 121–140. (10.1177/1088868319882596) 31642389

[17] von Scheve C , Ismer S . 2013 Towards a theory of collective emotions. Emot. Rev. **5** , 406–413. (10.1177/1754073913484170)

[18] Butler EA . 2011 Temporal interpersonal emotion systems: the 'TIES' that form relationships. Pers. Soc. Psychol. Rev. **15** , 367–393. (10.1177/1088868311411164) 21693670

[19] Dumas G , Fairhurst MT . 2021 Reciprocity and alignment: quantifying coupling in dynamic interactions. R. Soc. Open Sci. **8** , 210138. (10.1098/rsos.210138) 34040790 PMC8113897

[20] Siposova B , Carpenter M . 2019 A new look at joint attention and common knowledge. Cognition **189** , 260–274. (10.1016/j.cognition.2019.03.019) 31015079

[21] Shteynberg G . 2015 Shared attention. Perspect. Psychol. Sci. **10** , 579–590. (10.1177/1745691615589104) 26385997

[22] Wolf W , Launay J , Dunbar RIM . 2016 Joint attention, shared goals, and social bonding. Br. J. Psychol. **107** , 322–337. (10.1111/bjop.12144) 26256821 PMC4849556

[23] Wolf W , Tomasello M . 2020 Watching a video together creates social closeness between children and adults. J. Exp. Child Psychol. **189** , 104712. (10.1016/j.jecp.2019.104712) 31677423

[24] Wolf W , Tomasello M . 2020 Human children, but not great apes, become socially closer by sharing an experience in common ground. J. Exp. Child Psychol. **199** , 104930. (10.1016/j.jecp.2020.104930) 32693221

[25] Rennung M , Göritz AS . 2015 Facing sorrow as a group unites. Facing sorrow in a group divides. PLoS One **10** , e0136750. (10.1371/journal.pone.0136750) 26335924 PMC4559393

[26] Dunbar RIM , Teasdale B , Thompson J , Budelmann F , Duncan S , van Emde Boas E , Maguire L . 2016 Emotional arousal when watching drama increases pain threshold and social bonding. R. Soc. Open Sci. **3** , 160288. (10.1098/rsos.160288) 27703694 PMC5043313

[27] Dziura SL , Merchant JS , Alkire D , Rashid A , Shariq D , Moraczewski D , Redcay E . 2021 Effects of social and emotional context on neural activation and synchrony during movie viewing. Hum. Brain Mapp. **42** , 6053–6069. (10.1002/hbm.25669) 34558148 PMC8596971

[28] Chen KH , Brown CL , Wells JL , Rothwell ES , Otero MC , Levenson RW , Fredrickson BL . 2021 Physiological linkage during shared positive and shared negative emotion. J. Pers. Soc. Psychol. **121** , 1029–1056. (10.1037/pspi0000337) 32897091 PMC8261768

[29] Bruder M , Dosmukhambetova D , Nerb J , Manstead ASR . 2012 Emotional signals in nonverbal interaction: dyadic facilitation and convergence in expressions, appraisals, and feelings. Cogn. Emot. **26** , 480–502. (10.1080/02699931.2011.645280) 22471853

[30] Golland Y , Mevorach D , Levit-Binnun N . 2019 Affiliative zygomatic synchrony in co-present strangers. Sci. Rep. **9** , 3120. (10.1038/s41598-019-40060-4) 30816315 PMC6395718

[31] Cheong JH , Molani Z , Sadhukha S , Chang LJ . 2023 Synchronized affect in shared experiences strengthens social connection. Commun. Biol. **6** , 1099. (10.1038/s42003-023-05461-2) 37898664 PMC10613250

[32] Golland Y , Arzouan Y , Levit-Binnun N . 2015 The mere co-presence: synchronization of autonomic signals and emotional responses across co-present individuals not engaged in direct interaction. PLoS One **10** , e0125804. (10.1371/journal.pone.0125804) 26018597 PMC4446307

[33] Bizzego A , Azhari A , Campostrini N , Truzzi A , Ng LY , Gabrieli G , Bornstein MH , Setoh P , Esposito G . 2019 Strangers, friends, and lovers show different physiological synchrony in different emotional states. Behav. Sci. **10** , 11. (10.3390/bs10010011) 31877832 PMC7017247

[34] Kernan WN , Viscoli CM , Makuch RW , Brass LM , Horwitz RI . 1999 Stratified randomization for clinical trials. J. Clin. Epidemiol. **52** , 19–26. (10.1016/s0895-4356(98)00138-3) 9973070

[35] Bradley MM , Lang PJ . 1994 Measuring emotion: the self-assessment manikin and the semantic differential. J. Behav. Ther. Exp. Psychiatry **25** , 49–59. (10.1016/0005-7916(94)90063-9) 7962581

[36] MATLAB . 2019 9.7.0.1190202 (R2019b). Natick (MA), USA: The MathWorks Inc.

[37] Brainard DH . 1997 The Psychophysics Toolbox. Spat. Vis. **10** , 433–436. (10.1163/156856897X00357) 9176952

[38] Kleiner M , Brainard D , Pelli D , Ingling A , Murray R , Broussard C . 2007 What’s new in psychtoolbox-3? Percept. **36** , 1–16.

[39] Fraundorf SH *et al* . 2017 CogToolbox for MATLAB.

[40] Nakache O , Toledano E . 2011 The Intouchables. Clichy, France: Quad Films.

[41] Monson S . 2005 Earthlings. Malibu (CA), USA: Nation Earth.

[42] FreeHD videos – No Copyright . 2020 Online Education | Universities Library | Scholarship | Free Stock Footage [Video]. See https://youtu.be/PtLz0ZSKmBM?si=3vn44wuqtvIF3nKU.

[43] Gauthier J , Bouchard S . 1993 Adaptation canadienne-française de la forme révisée du state–trait anxiety inventory de spielberger. Can. J. Behav. Sci./Rev. can. des sci. du comport. **25** , 559–578. (10.1037/h0078881)

[44] Spielberger CD , Gorsuch RL , Lushene R , Vagg PR , Jacobs GA , Ed . 1983 Manual for the state-trait anxiety inventory. Palo Alto, CA: Spielberger.

[45] Coyne JC . 1976 Depression and the response of others. J. Abnorm. Psychol. **85** , 186–193. (10.1037//0021-843x.85.2.186) 1254779

[46] Cialdini RB , Brown SL , Lewis BP , Luce C , Neuberg SL . 1997 Reinterpreting the empathy-altruism relationship: when one into one equals oneness. J. Pers. Soc. Psychol. **73** , 481–494. (10.1037/0022-3514.73.3.481) 9294898

[47] Bouchat P , Pizarro JJ , Páez D , Zumeta LN , Basabe N , Włodarczyk A , Hatibovic F , Rimé B . 2024 Contributions of group identification and emotional synchrony in understanding collective gatherings: a meta-analysis of 13 studies. Gr. Process. Intergroup. Relat. (10.1177/13684302231223897)

[48] Kreibig SD . 2010 Autonomic nervous system activity in emotion: a review. Biol. Psychol. **84** , 394–421. (10.1016/j.biopsycho.2010.03.010) 20371374

[49] Bradley MM , Keil A , Lang PJ . 2012 Orienting and emotional perception: facilitation, attenuation, and interference. Front. Psychol. **3** , 493. (10.3389/fpsyg.2012.00493) 23181039 PMC3499912

[50] Boucsein W , Fowles DC , Grimnes S , Ben-Shakhar G , Roth WT , Dawson ME , Filion DL . 2012 Publication recommendations for electrodermal measurements. Psychophysiology **49** , 1017–1034. (10.1111/j.1469-8986.2012.01384.x) 22680988

[51] Grossman P , Taylor EW . 2007 Toward understanding respiratory sinus arrhythmia: relations to cardiac vagal tone, evolution and biobehavioral functions. Biol. Psychol. **74** , 263–285. (10.1016/j.biopsycho.2005.11.014) 17081672

[52] Oostenveld R , Fries P , Maris E , Schoffelen JM . 2011 FieldTrip: open source software for advanced analysis of MEG, EEG, and invasive electrophysiological data. Comput. Intell. Neurosci. **2011** , 156869. (10.1155/2011/156869) 21253357 PMC3021840

[53] Taylor S , Jaques N , Chen W , Fedor S , Sano A , Picard R . 2015 Automatic identification of artifacts in electrodermal activity data. Annual International Conference of the IEEE Engineeringin Medicine and Biology Society **2015** , 1934–1937. (10.1109/EMBC.2015.7318762) PMC5413200 26736662

[54] Grinsted A , Moore JC , Jevrejeva S . 2004 Application of the cross wavelet transform and wavelet coherence to geophysical time series. Nonlinear Process. Geophys. **11** , 561–566. (10.5194/npg-11-561-2004)

[55] Murata A , Nomura K , Watanabe J , Kumano S . 2021 Interpersonal physiological synchrony is associated with first person and third person subjective assessments of excitement during cooperative joint tasks. Sci. Rep. **11** , 12543. (10.1038/s41598-021-91831-x) 34131193 PMC8206359

[56] Dean RT , Dunsmuir WTM . 2016 Dangers and uses of cross-correlation in analyzing time series in perception, performance, movement, and neuroscience: the importance of constructing transfer function autoregressive models. Behav. Res. Methods **48** , 783–802. (10.3758/s13428-015-0611-2) 26100765

[57] Fujiwara K , Daibo I . 2018 Affect as an antecedent of synchrony: a spectrum analysis with wavelet transform. Q. J. Exp. Psychol. **71** , 2520–2530. (10.1177/1747021817745861)

[58] Sun Y , Greaves DA , Orgs G , de C. Hamilton AF , Day S , Ward JA . 2023 Using Wearable Sensors to Measure Interpersonal Synchrony in Actors and Audience Members During a Live Theatre Performance. Proc. ACM Interact. Mob. Wearable Ubiquitous Technol. **7** , 1–29. (10.1145/3580781)

[59] Danyluck C , Page-Gould E . 2019 Social and physiological context can affect the meaning of physiological synchrony. Sci. Rep. **9** , 8222. (10.1038/s41598-019-44667-5) 31160690 PMC6547677

[60] Palumbo RV , Marraccini ME , Weyandt LL , Wilder-Smith O , McGee HA , Liu S , Goodwin MS . 2017 Interpersonal autonomic physiology: a systematic review of the literature. Pers. Soc. Psychol. Rev. **21** , 99–141. (10.1177/1088868316628405) 26921410

[61] Laborde S , Mosley E , Thayer JF . 2017 Heart rate variability and cardiac vagal tone in psychophysiological research - recommendations for experiment planning, data analysis, and data reporting. Front. Psychol. **8** , 213. (10.3389/fpsyg.2017.00213) 28265249 PMC5316555

[62] R Core Team . 2022 *R: A language and environment for statistical computing*. Vienna, Austria: R Foundation for Statistical Computing.

[63] Team Rs . 2020 RStudio: integrated development for R. Boston, MA: RStudio, PBC.

[64] Bates D , Mächler M , Bolker B , Walker S . 2015 Fitting linear mixed-effects models using lme4. J. Stat. Softw. **67** , 1–48. (10.18637/jss.v067.i01)

[65] Christensen R . 2022 Package 'ordinal—regression models for ordinal data'. R package version 2022.11-16. [computer software].

[66] Afshartous D , Preston RA . 2011 Key Results of Interaction Models with Centering. J. Stat. Educ. **19** . (10.1080/10691898.2011.11889620)

[67] McNeish D . 2017 Small sample methods for multilevel modeling: a colloquial elucidation of REML and the Kenward-Roger correction. Multivariate Behav. Res. **52** , 661–670. (10.1080/00273171.2017.1344538) 28715244

[68] Stram DO , Lee JW . 1994 Variance components testing in the longitudinal mixed effects model. Biometrics **50** , 1171–1177. https://www.jstor.org/stable/2533455 7786999

[69] Lenth R , Singmann H , Love J , Buerkner P , Herve M . 2018 Package 'emmeans.' R Package Version 4.0-3 [computer software].

[70] Barr DJ . 2013 Random effects structure for testing interactions in linear mixed-effects models. Front. Psychol. **4** , 328. (10.3389/fpsyg.2013.00328) 23761778 PMC3672519

[71] Shapiro SS , Wilk MB . 1965 An analysis of variance test for normality (complete samples). Biometrika **52** , 591–611. (10.1093/biomet/52.3-4.591)

[72] Spearman C . 1904 The proof and measurement of association between two things. Am. J. Psychol. **15** , 72–10. (10.2307/1422689) 3322052

[73] Wilcoxon F . 1945 Individual comparisons by ranking methods. Biometrics Bull **1** , 80–83. (10.2307/3001968) 18903631

[74] Holm S . 1979 A simple sequentially rejective multiple test procedure. Scand. J. Stat. **6** , 65–70. https://www.jstor.org/stable/4615733

[75] Voeten C . 2022 Package 'buildmer': stepwise elimination and term reordering for mixed-effects regression. R package version 2.11. [computer software].

[76] Matuschek H , Kliegl R , Vasishth S , Baayen H , Bates D . 2017 Balancing Type I error and power in linear mixed models. J. Mem. Lang. **94** , 305–315. (10.1016/j.jml.2017.01.001)

[77] Bradley MM , Miccoli L , Escrig MA , Lang PJ . 2008 The pupil as a measure of emotional arousal and autonomic activation. Psychophysiology **45** , 602–607. (10.1111/j.1469-8986.2008.00654.x) 18282202 PMC3612940

[78] Palomba D , Sarlo M , Angrilli A , Mini A , Stegagno L . 2000 Cardiac responses associated with affective processing of unpleasant film stimuli. Int. J. Psychophysiol. **36** , 45–57. (10.1016/s0167-8760(99)00099-9) 10700622

[79] Shteynberg G , Hirsh JB , Apfelbaum EP , Larsen JT , Galinsky AD , Roese NJ . 2014 Feeling more together: group attention intensifies emotion. Emotion **14** , 1102–1114. (10.1037/a0037697) 25151520

[80] Rinck M , Primbs MA , Verpaalen IAM , Bijlstra G . 2022 Face masks impair facial emotion recognition and induce specific emotion confusions. Cogn. Res. Princ. Implic. **7** , 83. (10.1186/s41235-022-00430-5) 36065042 PMC9444085

[81] Dezecache G , Conty L , Chadwick M , Philip L , Soussignan R , Sperber D , Grèzes J . 2013 Evidence for unintentional emotional contagion beyond dyads. PLoS One **8** , e67371. (10.1371/journal.pone.0067371) 23840683 PMC3696100

[82] Jospe K , Genzer S , Klein Selle N , Ong D , Zaki J , Perry A . 2020 The contribution of linguistic and visual cues to physiological synchrony and empathic accuracy. Cortex. **132** , 296–308. (10.1016/j.cortex.2020.09.001) 33010739

[83] Schury VA , Nater UM , Häusser JA . 2020 The social curse: evidence for a moderating effect of shared social identity on contagious stress reactions. Psychoneuroendocrinology **122** , 104896. (10.1016/j.psyneuen.2020.104896) 33091760

[84] Wróbel M , Imbir KK . 2019 Broadening the perspective on emotional contagion and emotional mimicry: the correction hypothesis. Perspect. Psychol. Sci. **14** , 437–451. (10.1177/1745691618808523) 30844340

[85] Nasiopoulos E , Risko EF , Kingstone A . 2015 Social attention, social presence, and the dual function of gaze. In The many faces of social attention: behavioral and neural measures (eds A Puce , BI Bertenthal ), pp. 129–155. Cham, Switzerland: Springer. See 10.1007/978-3-319-21368-2_5.

[86] Baranowski-Pinto G , Profeta VLS , Newson M , Whitehouse H , Xygalatas D . 2022 Being in a crowd bonds people via physiological synchrony. Sci. Rep. **12** , 613. (10.1038/s41598-021-04548-2) 35022461 PMC8755740

[87] Tomashin A , Gordon I , Wallot S . 2022 Interpersonal physiological synchrony predicts group cohesion. Front. Hum. Neurosci. **16** , 903407. (10.3389/fnhum.2022.903407) 35903785 PMC9314573

[88] Zeevi L , Klein Selle N , Kellmann EL , Boiman G , Hart Y , Atzil S . 2022 Bio-behavioral synchrony is a potential mechanism for mate selection in humans. Sci. Rep. **12** , 4786. (10.1038/s41598-022-08582-6) 35314719 PMC8938461

[89] Mayo O , Lavidor M , Gordon I . 2021 Interpersonal autonomic nervous system synchrony and its association to relationship and performance - a systematic review and meta-analysis. Physiol. Behav. **235** , 113391. (10.1016/j.physbeh.2021.113391) 33744259

[90] Haj-Mohamadi P , Fles EH , Shteynberg G . 2018 When can shared attention increase affiliation? On the bonding effects of co-experienced belief affirmation. J. Exp. Soc. Psychol. **75** , 103–106. (10.1016/j.jesp.2017.11.007)

[91] Echterhoff G , Higgins ET , Levine JM . 2009 Shared reality: experiencing commonality with others’ inner states about the world. Perspect. Psychol. Sci. **4** , 496–521. (10.1111/j.1745-6924.2009.01161.x) 26162223

[92] Engelen T , Mennella R . 2023 Piecing together the puzzle of emotional consciousness. Neurosci. Conscious. **2023** , niad005. (10.1093/nc/niad005) 37034454 PMC10077334

[93] Noy L , Levit-Binun N , Golland Y . 2015 Being in the zone: physiological markers of togetherness in joint improvisation. Front. Hum. Neurosci. **9** , 187. (10.3389/fnhum.2015.00187) 25999832 PMC4419713

[94] Xygalatas D , Khan S , Lang M , Kundt R , Kundtová-Klocová E , Krátký J , Shaver J . 2019 Effects of extreme ritual practices on psychophysiological well-being. Curr. Anthropol. **60** , 699–707. (10.1086/705665)

[95] Whitehouse H . 2021 The ritual animal: imitation and cohesion in the evolution of social complexity. Oxford, UK: Oxford University Press.

[96] Kulik JA , Mahler HIM . 2000 Social comparison, affiliation, and emotional contagion under threat. In Handbook of social comparison. the springer series in social clinical psychology (eds J Suls , L Wheeler ). Boston, MA: Springer. (10.1007/978-1-4615-4237-7_15)

[97] Rimé B . 2009 Emotion Elicits the Social Sharing of Emotion: Theory and Empirical Review. Emot. Rev. **1** , 60–85. (10.1177/1754073908097189)

[98] Abeele MV , Roe K , Pandelaere M . 2007 Construct Validation of the Concepts Social Presence, Emotional Presence and Connectedness (ed. L Moreno ). In Proceedings of 10th international workshop on presence, Barcelona, Spain, October 25–27, 2007 pp. 215–224.

[99] Cho JC , Knowles ED . 2013 I’m like you and you’re like me: social projection and self-stereotyping both help explain self-other correspondence. J. Pers. Soc. Psychol. **104** , 444–456. (10.1037/a0031017) 23276270

[100] Osler L . 2020 Feeling togetherness online: a phenomenological sketch of online communal experiences. Phenomenol. Cogn. Sci. **19** , 569–588. (10.1007/s11097-019-09627-4)

[101] Holyst JA (ed). 2017 Cyberemotions: collective emotions in cyberspace. Cham, Switzerland: Springer.

[102] Stupacher J , Mikkelsen J , Vuust P . 2022 Higher empathy is associated with stronger social bonding when moving together with music. Psychol. Music **50** , 1511–1526. (10.1177/03057356211050681) 36097608 PMC9459360

[103] Rauchbauer B , Jank G , Dunbar RIM , Lamm C . 2023 Only empathy-related traits, not being mimicked or endorphin release, influence social closeness and prosocial behavior. Sci. Rep. **13** , 4072. (10.1038/s41598-023-30946-9) 36906682 PMC10008555

[104] Launay J , Tarr B , Dunbar RIM . 2016 Synchrony as an Adaptive Mechanism for Large‐Scale Human Social Bonding. Ethology **122** , 779–789. (10.1111/eth.12528)

[105] Hanich J . 2014 Watching a film with others: towards a theory of collective spectatorship. Scr. **55** , 338–359. (10.1093/screen/hju026)

[106] Pearce E , Launay J , Dunbar RIM . 2015 The ice-breaker effect: singing mediates fast social bonding. R. Soc. Open Sci. **2** , 150221. (10.1098/rsos.150221) 26587241 PMC4632513

[107] Lahnakoski JM , Forbes PAG , McCall C , Schilbach L . 2020 Unobtrusive tracking of interpersonal orienting and distance predicts the subjective quality of social interactions. R. Soc. Open Sci. **7** , 191815. (10.1098/rsos.191815) 32968493 PMC7481680

[108] Dale R , Bryant GA , Manson JH , Gervais MM . 2020 Body synchrony in triadic interaction. R. Soc. Open Sci. **7** , 200095. (10.1098/rsos.200095) 33047010 PMC7540751

[109] Thonhauser G . 2022 Towards a taxonomy of collective emotions. Emot. Rev. **14** , 31–42. (10.1177/17540739217540739)

[110] Spoor JR , Kelly JR . 2004 The evolutionary significance of affect in groups: communication and group bonding. Gr. Process. Intergroup Relat. **7** , 398–412. (10.1177/1368430204046145)

[111] Leary MR , Baumeister RF . 1995 The need to belong. Psychol. Bull. **117** , 497–529. (10.1037/0033-2909.117.3.497) 7777651

[112] Hirsch JL , Clark MS . 2019 Multiple paths to belonging that we should study together. Perspect. Psychol. Sci. **14** , 238–255. (10.1177/1745691618803629) 30517827

[113] Porat R , Halperin E , Mannheim I , Tamir M . 2016 Together we cry: social motives and preferences for group-based sadness. Cogn. Emot. **30** , 66–79. (10.1080/02699931.2015.1039495) 26016798

[114] Chung V , Mennella R , Pacherie E , Grezes J . 2024 Data from: Social bonding through shared experiences: the role of emotional intensity. Dryad Digital Repository. (10.5061/dryad.g79cnp5zf) PMC11521598 39479243

[115] Chung V , Mennella R , Pacherie E , Grezes J . 2024 Supplementary material from: Social bonding through shared experiences: the role of emotional intensity. Figshare. (10.6084/m9.figshare.c.7481225) PMC11521598 39479243

